# Laser speckle flowgraphy derived characteristics of optic nerve head perfusion in normal tension glaucoma and healthy individuals: a Pilot study

**DOI:** 10.1038/s41598-018-23149-0

**Published:** 2018-03-28

**Authors:** Anna Sophie Mursch-Edlmayr, Nikolaus Luft, Dominika Podkowinski, Michael Ring, Leopold Schmetterer, Matthias Bolz

**Affiliations:** 1 Kepler University Hospital Linz, Linz, Austria; 2 Singapore Eye Research Institute, Singapore National Eye Centre, Singapore, Singapore; 3 Department of Ophthalmology, Lee Kong Chian School of Medicine, Nanyang Technological University, Singapore, Singapore; 4 Ophthalmology and Visual Sciences Academic Clinical Program, Duke-NUS Medical School, Singapore, Singapore; 5 Department of Clinical Pharmacology, Medical University of Vienna, Vienna, Austria; 6 Center for Medical Physics and Biomedical Engineering, Medical University of Vienna, Vienna, Austria

## Abstract

The purpose of this prospective, case control study was to investigate the differences in optic nerve head blood flow measured with Laser Speckle Flowgraphy (LSFG) between Caucasian patients with normal tension glaucoma and healthy subjects. It included 20 eyes from 20 Caucasian patients with diagnosis of normal tension glaucoma and 20 eyes from age- and sex-matched healthy individuals. In the glaucoma group the antiglaucomatous therapy was paused 3 weeks prior to the investigations. Measurement of optic nerve head blood flow was performed with LSFG. The mean blur rate was obtained for different vascular compartments of the optic nerve head. Parameters for the characterization of pulse-waveform of the mean blur rate were calculated. It was shown that the mean blur rate was significantly lower in the glaucoma group compared to the control group (P < 0.001). The significant differences in the pulse-waveform parameters blow out time (P = 0.028) and flow acceleration time index (P < 0.001) indicate a flatter curve in NTG patients. In conclusion, LSFG can detect differences in optic nerve head blood flow between eyes with normal tension glaucoma and healthy eyes.

## Introduction

Glaucoma is a superordinate term that describes a group of multifactorial ophthalmic disorders that are characterized by progressive loss of retinal ganglion cells leading to typical optic nerve head (ONH) damage. Glaucoma is the second leading cause of irreversible blindness in the Western world^1^. Prevalence was estimated to increase to 79.6 million glaucoma patients in 2020 worldwide^2^. Glaucoma is characterized by an optic neuropathy with corresponding defects in the visual field. In primary open angle glaucoma an elevated intraocular (IOP) is considered the most important risk factor whereas in normal tension glaucoma (NTG) the damage of the optic nerve fibres occurs under statistically normal IOP. (≤21 mmHg)^1^. Even though the exact pathogenesis of NTG remains uncertain, it is understood that the disease develops as a consequence of several systemic and ocular factors^3^. There is some evidence that reduced optic nerve head microcirculation is associated with glaucoma progression^4,5^ but the exact relation between reduced blood flow and the disease process remains unknown^6,7^. Vascular factors seem to be important particularly in patients with normal-tension glaucoma^8–10^. Hence, non-invasive measurement of ocular perfusion and its pathophysiological alterations have been in the focus of intense research interest throughout the last decades^11,12^. Different methods are described in the literature (e.g. Laser Doppler Flowmetry), however, most of which require a high amount of examiner skill and patient compliance and are not available for the use in clinical practice^13^.

Laser speckle flowgraphy (LSFG) is a promising technique for the measurement of ocular perfusion. This method enables two-dimensional measurements of perfusion at the ONH, the retina and the choroid using the laser speckle phenomenon^14^. Developed in Japan, LSFG has gained widespread use in Japanese ophthalmic research facilities, but the feasibility in Caucasian healthy subjects was only demonstrated recently^13^.

Reports of Japanese NTG populations have endorsed LSFG as a promising tool for the screening of NTG^15^. In these reports, NTG patients were included irrespective of their current antiglaucomatous medication and, as of today, we are unaware of previous reports of LSFG in Caucasian glaucoma patients.

The purpose of the current study was to evaluate the difference in optic nerve head blood flow, determined by the LSFG-derived parameters of between Caucasian patients with untreated NTG and healthy individuals. The primary outcome parameter was the mean blur rate (MBR), which is a measure of erythrocytes velocity.

## Results

**Figure 1:**
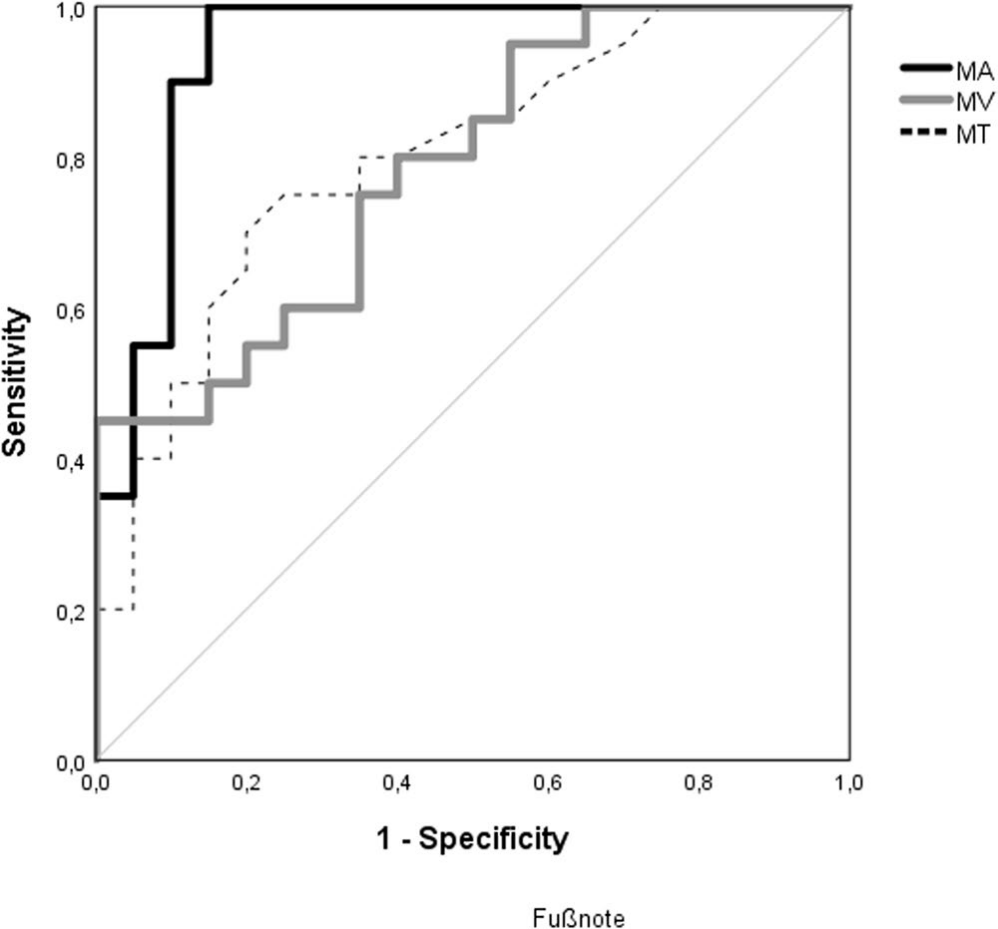
ROC curve for mean blur rate (MBR) in the whole optic nerve head area (MA), in the area of the big vessels (MV) and the microvasculature (MT). An area under the ROC curve (AUROC) of 1.0 represents perfect discrimination, whereas an AUROC of 0.5 can be considered chance discrimination.

Table 1 shows the baseline characteristics and the mean blur rate (MBR) in the three regions of optic nerve head (ONH) for the NTG group and the healthy control group. Significant differences between the groups were shown for the retinal nerve fibre layer (RNFL), MD and the MBR (MA, MV and MT). Table 2 shows the data of the waveform analysis of the MBR in the capillary area of the ONH. Data was adjusted for the heart rate. There was a significant difference in the parameter blow out time with higher values in the NTG group. The parameter flow acceleration index was significantly lower in patients with NTG. In the NTG group MBR showed a positive correlation with the RNFL in all areas. We could show a statistical trend for MA but it did not reach statistical significance. (MA, r = 0.458, p = 0.042; MV, r = 0.396, p = 0.084; MT, r = 0.362; p = 0.12). A ROC curve for the MBR in its regions is shown in Fig. 1. AUC was 0.94 for MA, 0.79 for MT and 0.8 for MV. Sensitivity at 90% specificity is shown in Table 3.

**Table 1:** Baseline characteristics and Mean Blur Rate in normal tension glaucoma group and control group including biometric results. Glaucomatous damage was graded into groups according to the visual field impairment^30^.

Characteristics		NTG Number, mean ± SD	Control group Number, mean ± SD	P Value
Number		20	20	
Sex	Male	7	7	
	Female	13	13	
Age (years)		70.9 ± 9.1	71.0 ± 8.5	0.95
SBP (mmHg)		144.0 ± 11.3	145.2 ± 11.6	0.73
DBP (mmHg)		81.8 ± 8.0	78.0 ± 7.9	0.14
HR (bpm)		69.7 ± 13.4	72.6 ± 10.5	0.45
MRSE (dpt)		−0.7 ± 1.5	−0.4 ± 1. 1	0.46
BCVA (LogMAR)		0.05 ± 0.07	0.03 ± 0.01	0.6
AGDs		1.3 ± 0.73	—	
	PGA	16 (80%)		
	PGA + BB	3 (15%)		
	PGA + BB + AA + CAI	1 (5%)		
IOP (mmHg)	Under therapy	12.85 ± 1.9		
	No therapy (After washout)	15.3 ± 2.2	15.6 ± 2.6	0.8
MD (dB)		−9.2 ± 6.5	−1.46 ± 1.37	< 0.001*
Glaucoma grade	Mild	7 (35%)		
	Moderate	6 (30%)		
	Severe	4 (20%)		
	NA	3 (15%)		
cp RNFL(μm)		67.0 ± 8.5	95.5 ± 9.0	< 0.001*
MBR (AU)	MA	11.36 ± 2.8	16.9 ± 2.9	< 0.001*
	MV	24.9 ± 5.7	31.6 ± 5.6	0.001*
	MT	6.7 ± 1.6	8.9 ± 2.0	0.001*

(NTG) Normal tension glaucoma. (SD) Standard deviation. (MBR) Mean blur rate. (SBP) Systolic blood pressure. (DBP) Diastolic blood pressure. (HR) Heart rate. (bpm) Beats per minute. (BCVA) Best corrected visual acuity. (AGDs) Antiglaucomatous drugs. (PGA) Prostaglandin-analogue monotherapy. (PGA + BB) Combination of prostaglandin analogue and beta blocker. (PGA + BB + AA + CAI) Combination of prostaglandin analogue, beta blocker, alpha agonist and carbonic anhydrase inhibitor. (IOP) Intraocular pressure. (MD) Mean deviation of visual field. (NA) Not applicable. (cpRNFL) Circumpapillary retinal nerve fibre layer. (AU) Aberration units. (MA) MBR in all area of interest. (MV) MBR in area of big vessels. (MT) MBR in area of microvasculature. (*Marks statistical significance).

**Table 2:** Differences in waveform parameters describing the curve of mean blur rate in the area of the capillaries (MT).

Waveform parameters	NTG mean ± SD	Control group mean ± SD	P Value
BOT	45.83 ± 3.95	43.22 ± 3.21	0.028*
BOS	71.83 ± 6.31	68.52 ± 5.06	0.075
Skew	13.32 ± 1.86	14.05 ± 2.48	0.3
ATI	31.79 ± 3.48	32.45 ± 2.85	0.518
RR	12.32 ± 0.79	12.13 ± 0.71	0.427
FR	13.45 ± 4.43	14.79 ± 3.21	0.28
FAI	0.56 ± 0.18	0.84 ± 0.27	0.001*
RI	0.41 ± 0.07	0.45 ± 0.05	0.026
Fluctuation	15.94 ± 3.59	18.17 ± 3.31	0.048

(NTG) Normal tension glaucoma. (SD) Standard deviation. (BOT) Blow out time. (BOS) Blow out score. (ATI) Acceleration time index. (RR) Rising rate. (FR) Falling rate. (FAI) Flow acceleration index. (RI) Resistivity index. Data were adjusted for the heart rate. (*Marks statistical significance).

**Table 3:** Sensitivity of mean blur rate in all compartments at 95% specificity.

	Sensitivity (%)	Cut-off
MA	85	13.9
MV	45	31.6
MT	45	8.8

(MA) Mean blur rate in all area of interest. (MV) Mean blur rate in area of big vessels. (MT) Mean blur rate in area of microvasculature.

## Discussion

The aim of this study was to assess the optic nerve head (ONH) blood flow in patients with normal tension glaucoma (NTG) by laser speckle flowgraphy (LSFG). It has previously been described elsewhere that LSFG shows high success rates and repeatability in Caucasian subjects^13^ and its reliability exceeds the numbers described for scanning laser Doppler flowmetry (LDF reliability coefficient for flow at OHN rim 0.87 versus 0.98 for MA in LSFG)^13,16^. There are several differences between LDF and LSFG that have been discussed in details recently^17^. As compared to scanning LDF the laser is not scanned over the retina, thereby avoiding that the movement of the laser beam over the tissue by itself causes a Doppler shift^18^. To our knowledge, the present work is the first to apply this non-invasive technique in a cohort of Caucasian NTG patients. In the present study we showed significant differences in ONH blood flow in NTG patients, compared to a healthy, age- and sex-matched control group. The mean blur rate (MBR, MA) and values derived from it (MV, MT) were significantly lower in patients with NTG, which we see in concordance with data previously published for Japanese patients with NTG^19–21^. The reduction of MV can be interpreted as reduced blood flow in the vessels supplying the retina. It has been described elsewhere that narrowing of the retinal arteries can be considered a risk factor for developing glaucoma^22^. Analysis of the waveform of the MBR in patients with NTG also revealed significant differences compared to the control group. The parameter blow out time is calculated from the ratio of the half-width of the pulse-waveform curve and the total duration of the cardiac cycle. It showed significantly higher values in the glaucoma group in the present study. In the literature a high blow out time is interpreted as a marker for well-maintained perfusion for a long period of time during each heartbeat and is known to decrease with age^13^. Considering the formula for calculating blow out time, however, one will become aware that high blow out time does not necessarily indicate a sustained period of elevated MBR levels but can also be caused by a low maximum MBR. We thus hypothesize that the high blow out time in patients with NTG indicates a flatter curve with less MBR max than in healthy subjects. We see this hypothesize in accordance with the finding that flow acceleration index is significantly lower in NTG patients, indicating a smaller maximum steepness of the ascending part between two frames of the pulse-waveform curve. Other parameters like ATI or skew did not differ significantly, indicating that the orientation of the curve and the time to reach the peak were comparable between the groups. Our findings on the waveform parameters compete with another study in the literature where 61 eyes with NTG were compared to 21 healthy control subjects^15^. Authors analyzed exclusively the parameters skew, blow out time and acceleration time index and report that skew was significantly lower and acceleration time index significantly higher in the glaucoma group. No significant difference was shown regarding blow out time but the authors do conclude that they would have expected a negative correlation and therefore indication of a flattened waveform with advanced glaucoma grade. Differences in the outcomes mainly could be due to the fact, that in the aforementioned study data were analysed retrospectively and patients were on different numbers and classes of glaucoma medications whereas subjects in the present work had stopped all antiglaucomatous drugs before the LSFG measurement. Herein we therefor minimized a possible influence from therapy on ONH BF, which was shown in other studies before^13,23^. Differences may be also related to the fact that the study subjects from the current work showed different degrees of glaucomatous damage, and Shiga *et al*. showed that changes in waveform parameters depend on the glaucoma stage^15^.

However, the sample size in the current study was small which limited further subgroup analysis. Also Correlating LSFG parameters with RNFL thickness indicated a positive relation for all MBR parameters. However, we only showed a statistical trend for MA, which did not reach the predefined level of statistical significance. This weak correlation between blood flow and structure mainly can be explained by the small sample size. However we see our result in accordance with the literature^19^. In the ROC curve analysis we showed that MA is a potential biomarker to differentiate NTG from healthy eyes. Other studies analysed different LSFG parameters for their screening potential and found e.g. an AUC for skew of 0.89 and for acceleration time index of 0.8^15,24^. Thus, compared to these findings MA has a strong predictive value for differentiation between NTG and healthy eyes with excellent sensitivity and specificity.

Limitation to this work may be found. Patients were included in the NTG group if there was no history of intraocular pressure (IOP) exceeding 21 mmHg but we did not confirm this assumption by a diurnal IOP profile. A sample size of 20 eyes with NTG limits the result. Normal tension glaucoma is less common in Europe compared to Asia^25^ and as this study was planned as a pilot study we limited the sample size to 20 patients. Though we did not assess it directly we considered a washout period of three weeks sufficient, considering the literature on pharmacokinetic of latanoprost and timolol^26,27^. Due to the cross-sectional nature of the study it remains unclear whether the impaired blood flow is cause or consequence of the disease. However, we consider the study design superior compared to the abovementioned studies as it was conducted in a prospective-controlled setting.

In conclusion, the present study demonstrated significant differences in ONH blood flow in NTG patients compared to healthy subjects. Besides a significant reduction in MBR in all ONH blood flow compartments we also revealed a deviation of the waveform, indicating a flattened curve with less MBR max in eyes with NTG. Our findings concerning a positive correlation between ONH blood flow and RNFL, are in agreement with earlier studies^19^ but did not reach statistical significance in the small study group. Further prospective, longitudinal studies are required to determine whether the observed changes in ONH blood flow are crucial in the pathophysiology of NTG, or whether they result as a secondary phenomenon due to retinal ganglion cell loss leading to a decreased metabolic demand for maintaining perfusion^8^. LSFG can be a useful tool in these future evaluations.

## Methods

This prospective, cross-sectional pilot study included 20 eyes of 20 Caucasian adult patients with NTG and 20 eyes of 20 healthy age and sex-matched control subjects. NTG patients were recruited consecutively from the glaucoma clinic of the Kepler University Hospital Linz, Austria. Patients were either asked to participate in the trial when they came for regular follow up or were called and invited for a screening visit when they appeared as potential study subjects in the files. Subjects from the control group were healthy volunteers. They were employees of the clinic or have been treated for other ophthalmic diseases (e.g. dry eye, cataract) in the Kepler University Hospital. They were asked to join the trial if there was no sign of glaucoma and subjects fitted according sex and age. The study protocol was reviewed and approved by the local ethics committee (Ethikkommission des Landes Oberösterreichs) and followed the guidelines set forth in the Declaration of Helsinki. Written informed consent was obtained before inclusion in the study. All subjects underwent a comprehensive screening examination, including a slit-lamp examination with indirect funduscopy and measurement of intraocular pressure (IOP) using Goldmann applanation tonometry. Indirect gonioscopy was performed, and patients with narrow angles or other angle abnormalities were excluded (based on Shaffer grading^1^). Best-corrected visual acuity (BCVA) was assessed using the Jackson cross-cylinder method and the standard ETDRS visual acuity chart.

The inclusion criteria in the NTG group were (1) presence of glaucomatous optic disc changes in biomicroscopy and visual field defects or abnormal circumpapillary retinal nerve fibre layer (RNFL) thinning (evaluated by Cirrus OCT as defined by the on board software), (2) IOP ≤ 21 mmHg without therapy, (3) normal open angle in a gonioscopic examination, (4) age > 40 years. All patients with NTG were diagnosed at the investigation site. The exclusion criteria were (1) IOP > 21 mmHg in patients history, (2) ametropia > 6 diopters, (3) cataract with severity greater than grade 2 of the lens opacities classification system (LOCS) classification, (4) the presence of corneal opacities, (5) ocular surgery within 3 months prior to participation in the study, and (6) uncontrolled hypertension with systolic blood pressure (SBP) > 160 mmHg and/or diastolic blood pressure (DBP) > 100 mmHg.

Patients with NTG included in the study were instructed to stop their antiglaucomatous therapy, followed by weekly IOP measurements to detect a rise of IOP over 21 mmHg. Measurements were conducted after a washout period of 3 weeks. If both eyes of a subject were eligible, the eye with greater glaucomatous damage, defined by the mean deviation (MD) in visual field testing and/or RNFL thickness was selected as study eye.

Subjects were instructed to abstain from alcohol and stimulating beverages containing xanthine derivatives (e.g. tea, coffee) 12 hours before the laser speckle flowgraphy (LSFG) measurements, as these are known to potentially influence the results^28^. Measurements were performed in a quiet, dark room with the subject in sitting position.

### Visual field

Visual field testing was performed with the Humphrey HFA 745i perimeter (Carl Zeiss Meditec AG, Jena, Germany). Patients were tested with the 30–2 program, using the Swedish interactive threshold algorithm (SITA) standard strategy. Mean deviation was recorded as an objective parameter. Only reliable MD values were used, excluding examinations with 20% fixation errors and <33% false- positives or false-negatives^19,25,29^. Study eyes from the NTG group were graded into groups according to the visual field impairment: mild (MD > −6 dB), moderate (MD between −6 and −12 dB and severe (MD < −12 dB) glaucomatous damage^30^.

### Retinal nerve fibre layer thickness (RNFL)

The RNFL was measured with a commercially available spectral domain optical coherence tomography (SD-OCT) system (Cirrus, Carl Zeiss AG, Germany). After obtaining the circumpapillary scan the software automatically calculates the average RNFL thickness.

### Laser speckle flowgraphy

**Figure 2:**
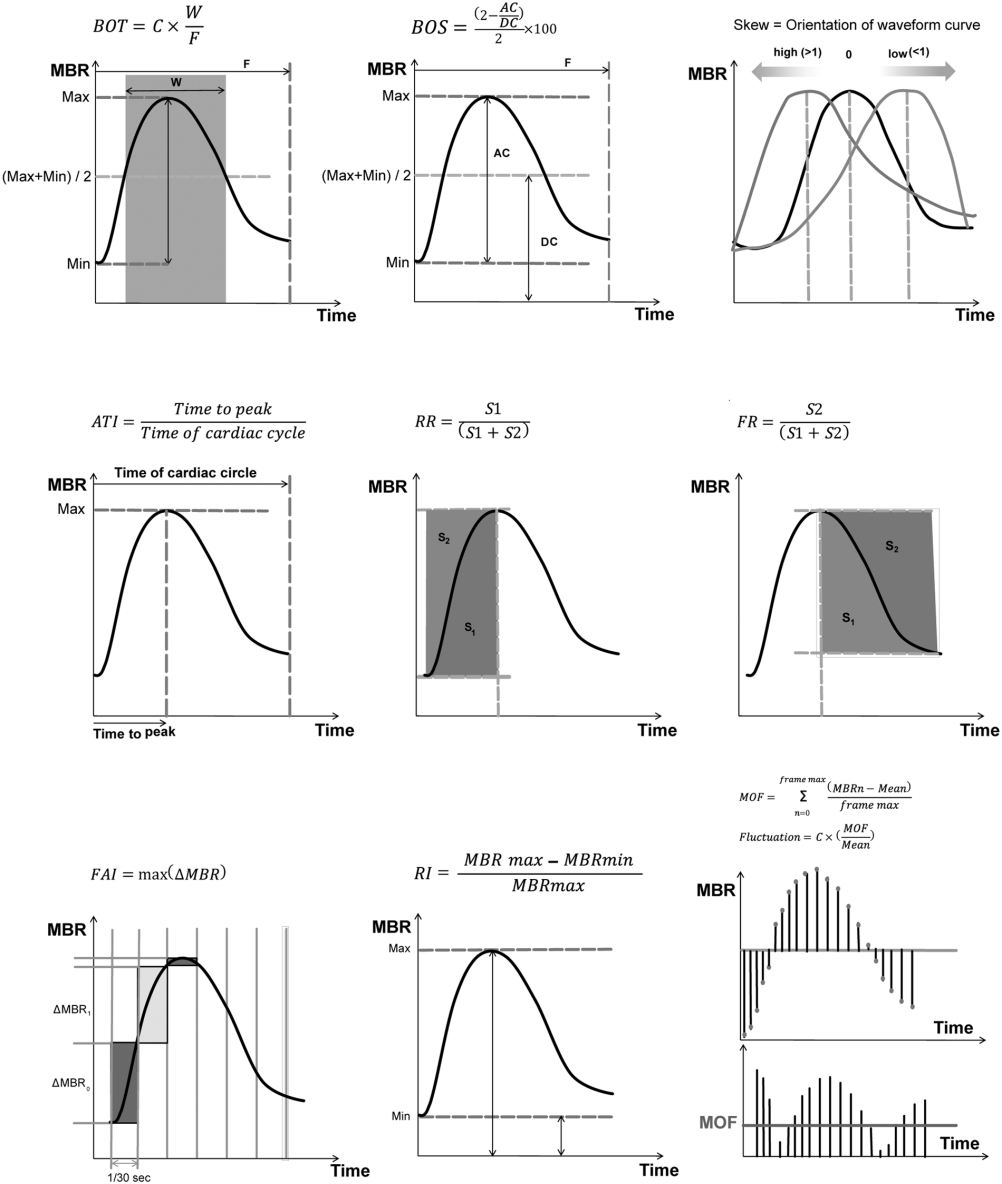
Calculation of the pulse-waveform parameters. Top left: Blow out time (BOT). Top center: blowout score (BOS). Top right: skew. Middle left: acceleration time index (ATI). Middle center: rising rate (RR). Middle right: falling rate (FR). Bottom left: the flow acceleration index (FAI). Bottom center: resistivity index (RI). Bottom right: fluctuation, (MOF) mean of fluctuation.

LSFG measurements were performed out with the LSFG-NAVI instrument (Softcare Co., Ltd., Fukuoka, Japan) after pharmacological dilation of the pupil with 0.5% tropicamide eye drops (Mydriaticum Agepha Augentropfen; Agepha Ges.m.b.H., Vienna, Austria). The LSFG device consists of a fundus camera equipped with a diode laser at a wavelength of 830 nm and a digital charge-coupled device camera (750 × 360 pixels). A total of 118 images are acquired at a rate of 30 frames per second over a 4-second measurement period. The primary output parameter of LSFG for the quantification of perfusion at the optic nerve head (ONH) is the mean blur rate (MBR). It is a quantitative index of retinal blood cell (RBC) velocity, and thereby a measurement of the relative blood flow. MBR was calculated for the total ONH area (referred to as MA, “mean MBR of all area”), for the large vessels within the ONH (MV, “mean MBR of vascular area”) and the tissue area containing the microvasculature (MT, “mean MBR of tissue area”). With the current software version various parameters characterizing the shape of the MBR waveform at each BF compartment (MA, MV or MT) during one cardiac cycle can be calculated (“pulse-waveform analysis”). The parameter blow out time (BOT) is the ratio of the half width (the time that the MBR is higher than half of the mean of the minimum and maximum signal) to the duration of one complete cardiac cycle. Blowout score (BOS) is calculated from the difference of the maximum and the minimum MBR as well as the average MBR. Skew indicates the asymmetry of the waveform distribution, whereas 0 describes a perfectly symmetrical waveform shape. A leftward distribution is described by a positive value. Skew increases with a steeper decline of the waveform curve after the peak indicating a more rapid drop-off in blood flow after the peak. Acceleration time index (ATI) is defined as the ratio of time to reach the pulse-wave peak and the duration of the entire heartbeat. Rising rate (RR) and falling rate (FR) describe the steepness of the ascending and, respectively, descending part of the curve. Higher values indicate a more sudden increase, or decrease, of MBR. The flow acceleration index (FAI) shows the highest increment of MBR between two frames. The resistivity index (RI) is calculated from the ratio of the difference between maximum and minimum MBR to the maximum MBR. Fluctuation describes the dimension of the amplitude of the waveform curve. The principles of the equations for the calculation of these parameters are shown in Fig. 2.

### Statistics

Statistical analyses were conducted using SPSS software version 21.0 (SPSS Inc., Chicago, IL, USA). Descriptive data are presented as mean and standard deviation. At first, histogram frequency analysis and the Shapiro-Wilk test was performed to confirm the normal distribution of data. Comparison of means between study and control group was performed with the Student’s t-Test for unpaired samples. To compensate for multiple testing we applied the Bejamini-Hochberg procedure. Correlation analyses with calculation of Pearson correlation coefficient was used to determine the correlation between the RNFL thickness and the LSFG parameters. A p value of <0.05 was considered statistically significant. Receiver operating characteristic (ROC) curves were used to describe the diagnostic ability of MBR parameters to differentiate NTG from normal eyes. The datasets generated during the current study are available from the corresponding author on reasonable request.
